# Circulating MicroRNAs in the Second Trimester From Pregnant Women Who Subsequently Developed Preeclampsia: Potential Candidates as Predictive Biomarkers and Pathway Analysis for Target Genes of miR-204-5p

**DOI:** 10.3389/fphys.2021.678184

**Published:** 2021-09-22

**Authors:** Marcelo R. Luizon, Izabela M. C. A. Conceição, Sarah Viana-Mattioli, Mayara Caldeira-Dias, Ricardo C. Cavalli, Valeria C. Sandrim

**Affiliations:** ^1^Department of Genetics, Ecology and Evolution, Institute of Biological Sciences, Federal University of Minas Gerais, Belo Horizonte, Minas Gerais, Brazil; ^2^Department of Biophysics and Pharmacology, Institute of Biosciences, Universidade Estadual Paulista (UNESP), Botucatu, São Paulo, Brazil; ^3^Department of Gynecology and Obstetrics, Ribeirao Preto Medical School, University of São Paulo, Ribeirao Preto, São Paulo, Brazil

**Keywords:** biomarkers, microRNAs, preeclampsia, pregnancy, gene expression profiling, gene expression regulation, signaling pathways

## Abstract

MicroRNAs (miRNAs) play an important role in the pathophysiology of preeclampsia (PE). However, the expression of circulating miRNAs was not analyzed in the second trimester of pregnancy, a period of major relevance to identify predictive biomarkers for PE. Therefore, we examined the expression profiles of 84 circulating miRNAs using a PCR array in plasma collected between 20 and 25 weeks of gestation from pregnant women, who subsequently developed PE and those who remained healthy during pregnancy, randomly selected from a prospective cohort. Overall, 23 miRNAs had a fold change > 2.0 and were considered to be upregulated in plasma from pregnant women who subsequently developed PE, even before the onset of clinical symptoms of PE. However, only miR-204-5p was statistically significant (*P* = 0.0082). Experimentally validated interactions for the target genes of miR-204-5p extracted from miRTarBase were used in the gene set functional enrichment analysis to identify Reactome pathways. The network connecting the 37 target genes for miR-204-5p revealed pathways of known pathophysiological relevance during the early development of PE and included key genes related to PE, such as *BDNF, MMP-9, MALAT1, TGFBR2*, and *SIRT1*. We further depicted downstream targets of SIRT1 that are related to the vascular endothelial function or implicated in the pathophysiology of PE, namely, FOXO1, NFκB, HIF-1α, NOS3, and PPAR-γ. Our novel findings provide for circulating miRNAs upregulated in the second trimester on plasma from pregnant women who subsequently developed PE that is potentially related to the early development of PE, which may guide further studies focused on the validation of potential predictive biomarkers in PE.

## Introduction

Preeclampsia (PE) is characterized by hypertension after 20 weeks of gestation, which may be accompanied by proteinuria or thrombocytopenia, renal insufficiency, impaired liver function, pulmonary edema, or cerebral/visual symptoms (American College of Obstetricians Gynecologists Pregnancy TFOHI, 2013). PE affects up to 9% of all pregnancies, it is the major cause of maternal and fetal morbidity and mortality worldwide, and the only definitive treatment is the delivery of the placenta (Umesawa and Kobashi, 2017; Rana et al., 2019). Despite its burden, PE is a multisystem syndrome, and its pathophysiology is complex and not fully elucidated, which limits current management therapies.

MicroRNAs (miRNAs) are short endogenous noncoding RNA transcripts of 18–24 nucleotides that posttranscriptionally regulate gene expression by either degradation or translation repression (Bartel, 2009). It is well known that miRNAs may play an important role in the pathophysiology of PE (Sandrim et al., 2016; Bounds et al., 2017; Caldeira-Dias et al., 2018; Lv et al., 2019; Skalis et al., 2019). Specifically, it has been shown that even before the onset of clinical symptoms of PE, the expression of circulating miRNAs in the first trimester of pregnancy was altered in pregnant women who later developed PE in the third trimester as compared with those who remained healthy during pregnancy (Luque et al., 2014; Hromadnikova et al., 2017, 2019). Since circulating miRNAs are very stable, they have been proved as useful biomarkers for several disorders, including cancer, cardiovascular, and immunoinflammatory diseases (De Guire et al., 2013).

Several studies have focused on identifying specific biomarkers for the early prediction of PE, including miRNAs (Jadli et al., 2015; Lv et al., 2019). To our knowledge, three studies have examined plasma collected in the first trimester of pregnancy to evaluate the deregulated expression of miRNAs as predictors of PE (Luque et al., 2014; Hromadnikova et al., 2017, 2019). The aim to identify biomarkers in the first trimester of pregnancy is to start pharmacological prophylaxis with the use of aspirin and calcium in high-risk women. However, no previous study has analyzed the expression of circulating miRNAs in the second trimester of pregnancy, a period of major importance to identify predictive biomarkers to increase the monitoring of pregnancy, because most of the cases of PE develop after 25 weeks of gestation. Therefore, we aimed to perform a miRNA screening on plasma collected between 20 and 25 weeks of gestation from pregnant women who subsequently developed PE in order to search for differentially expressed miRNAs and provide for potential candidates as predictive biomarkers in PE.

In this study, we examined the expression profiles of 84 circulating miRNAs using a PCR array in plasma collected in the second trimester from pregnant women who subsequently developed PE and compared with those who remained healthy during pregnancy. Moreover, we extracted the target genes for the upregulated miR-204-5p (fold change > 2.4; *P* = 0.0082) from the miRTarBase of experimentally validated miRNA-target interactions and used in the gene set functional enrichment analysis to identify Reactome pathways potentially deregulated in the pathophysiology of PE.

## Methods

### Subjects

**Figure 1:**
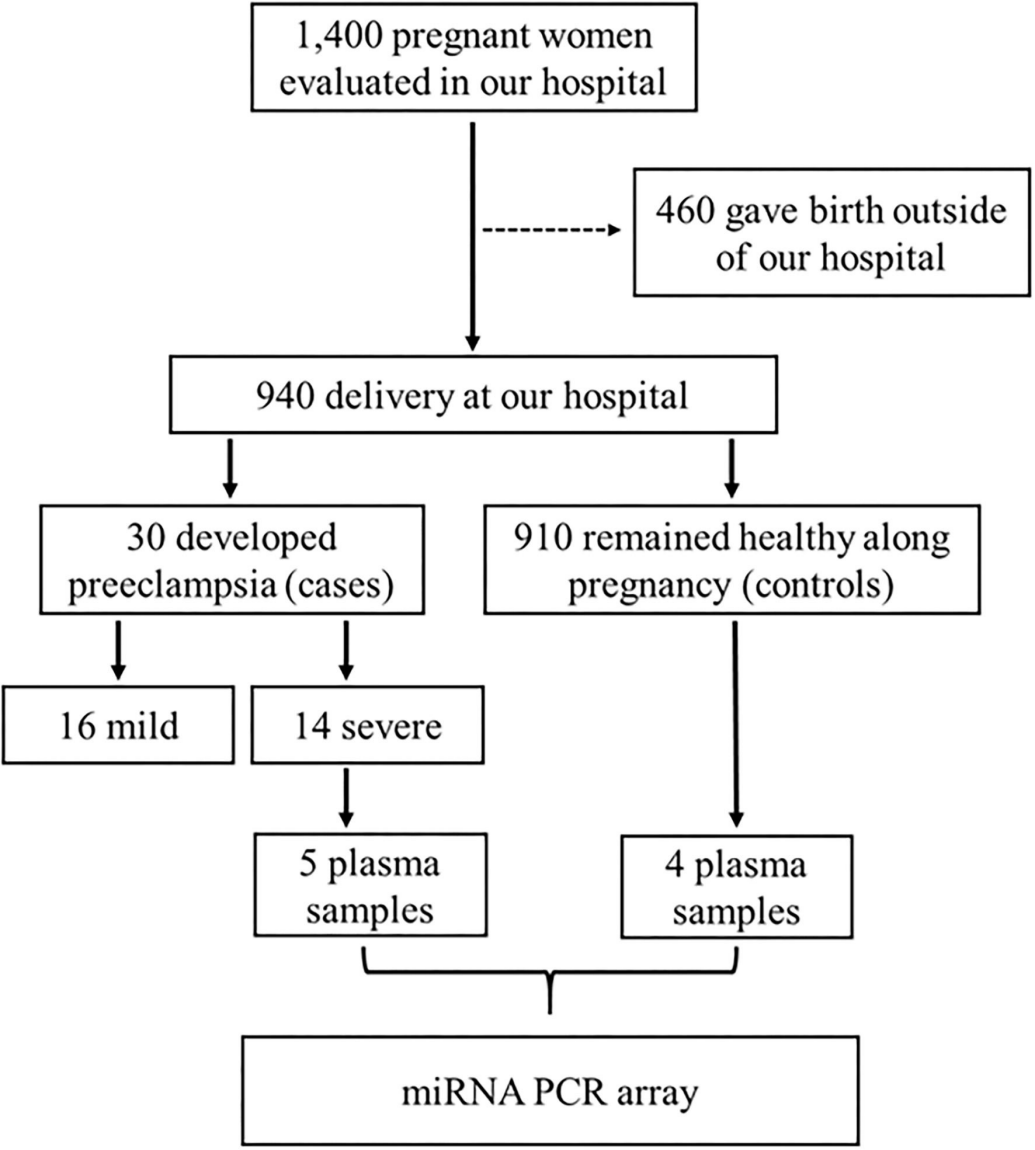
Schematic diagram of the study workflow for the inclusion of subjects, and the selection of plasma samples from pregnant women who developed severe preeclampsia (case group) or those who remained healthy during pregnancy (control group), which were used in the PCR array. This case-control study is based in a prospective cohort named Brazilian Ribeirão Preto and São Luís prenatal cohort (BRISA) (da Silva et al., 2014; Pereira et al., 2016; Caldeira-Dias et al., 2019).

The study was approved by the Institutional Review Board of the University of São Paulo at Ribeirão Preto (reference 4116/2008), according to the declaration of Helsinki, and all participants provided written informed consent. This case-control study is based in a prospective cohort, Brazilian Ribeirão Preto and São Luís prenatal cohort (BRISA) (da Silva et al., 2014; Pereira et al., 2016; Caldeira-Dias et al., 2019). The schematic diagram of the study workflow is shown in Figure 1.

Pregnant women (*n* = 1,400) with gestational age between 20 and 25 weeks from our prospective cohort were evaluated at the *Hospital das Cl*í*nicas de Ribeirão Preto* at the University of São Paulo (prenatal cohort), but 460 pregnant women gave birth outside the institution. Of the remaining 940 pregnant women, 30 subsequently developed PE and 910 remained healthy during pregnancy (control group). PE was defined as pregnancy-induced hypertension (≥ 140 mmHg systolic and ≥ 90 mmHg diastolic on two or more measurements, at least 6 h apart) in a woman after 20 weeks of gestation, and returning to normal by 12 weeks postpartum, and significant proteinuria (≥ 0.3 g/24 h), and it was further classified into mild (*n* = 16) or severe (*n* = 14) according to the American College of Obstetricians and Gynecologists (American College of Obstetricians Gynecologists Pregnancy TFOHI, 2013). Plasma samples from pregnant women with severe PE (*n* = 5, case group) and from the control group (*n* = 4) were randomly selected for this study (Figure 1).

Maternal venous blood samples were collected in Vacutainer tubes (Becton-Dickinson, São Paulo, Brazil) using EDTA as an anticoagulant and centrifuged at room temperature. Plasma samples were stored at −80°C until analysis.

### Total RNA Isolation and mRNA Expression Using PCR Array

Total RNA from plasma samples (200 μl) from control and case groups was isolated using the miRNeasy Serum/Plasma Kit (Qiagen®, Leusden, Netherlands) according to the instructions of the manufacturer. Noteworthy, we have performed several steps to ensure the quality of the miRNA expression results, which included the calibration of miRNA extraction using cel-miR-39 and the normalization using commonly expressed miRNA targets, as previously reported (Sandrim et al., 2016). Synthetic *Caenorhabditis elegans* cel-miR-39 (#219610, Qiagen®) was added at known amounts during the isolation in each biological sample in order to estimate the efficiency of RNA extraction and reverse transcription (RT) reaction.

The Human Serum & Plasma miScript miRNA PCR Array (MIHS-106Z, Qiagen®, Leusden, Netherlands) was used to evaluate the expression of 84 miRNAs detectable in plasma, as well as seven different small RNAs that were used as normalization control. The PCR array also contained replicate reverse transcription and positive controls.

For the PCR array, the total RNA isolated was converted into cDNA using the miScript II RT Kit (Qiagen®, Leusden, Netherlands), according to the instructions of the manufacturer, which contains a buffer that selectively converts mature miRNAs, certain small nucleolar RNAs, and small nuclear RNAs into cDNA. qPCR was performed using 1375 μl of 2 × QuantiTect SYBR Green PCR Master Mix, 275 μl of 10 × miScript Universal Primer, 1000 μl of RNase-free water, and 100 μl of cDNA (1.5 ng/μl) from each sample in a final volume of 2750 μl per plate. Thermal cycling was performed using the following conditions: 15 min at 95°C, 40 two-step cycles of 15 s at 94°C, 30 s at 55°C, 30 s at 70°C, and a final step for the dissociation curve. The C_T_ (cycle threshold) is the number of cycles required for the fluorescent signal to cross the threshold.

Normalization was performed using the SNORD95, SNORD96A, RNU6-6P, and cel-miR-39 from the housekeeping (HKG) panel in the array, as they were the most stable genes in our samples. The cel-miR-39 was measured concomitantly to the miRNAs of the PCR array. After obtaining the C_T_ value of each sample, ΔC_T_ was determined by the equation ΔC_T_ = C_T_sample – Average C_T_HKG. Then, ΔΔC_T_ was calculated by the equation ΔΔC_T_ = ΔC_T_PE – ΔC_T_HP. The fold change in miRNA expression was calculated using the comparative 2(–ΔΔC_T_) method (Livak and Schmittgen, 2001).

### Statistical and miRNA Expression Data Analyses

Clinical characteristics between pregnant women who subsequently developed PE during pregnancy (case group) and those who remained healthy during pregnancy (control group) were compared using the Student's *t*-tests, which were performed using GraphPad Prism 5.0 Software (San Diego, CA, USA).

The QIAGEN's GeneGlobe Data Analysis Center (https://geneglobe.qiagen.com/us/analyze/) online platform was used to analyze the miRNA expression data. Briefly, the CT values for the PCR arrays with individual plasma samples from the case and the control groups were exported to an Excel file and uploaded into the GeneGlobe Data Analysis Center, which calculated the fold change using the 2(–ΔΔC_T_) method. The *P-*values were calculated based on the Student's *t*-test of the replicate 2 ^(−Δ*CT*)^ values for each gene in the case and the control groups. A value of fold change > 2.0 in the case compared with control groups was the cutoff value used to consider the expression of miRNAs as upregulated (Zhao et al., 2018). For all tests, a *P* < 0.05 (two-tailed test) was considered significant.

### Experimentally Validated Interactions for Target Genes of miR-204-5p From miRTarBase

Target genes of the upregulated miR-204-5p were extracted from the selection of experimentally validated miRNA-target interactions in *Homo sapiens* included in the miRTarBase database (Huang et al., 2020). The miRTarBase contains manually curated information from research articles that actually performed the experiments to validate the target genes of miR-204-5p, which are referenced by the respective PubMed ID (PMID; Supplementary Table 1). Notably, almost all experiments were luciferase reporter assays, along with qRT-PCR, western blot, and/or immunohistochemistry (Supplementary Table 1).

### Gene Set Functional Enrichment Analysis and Reactome Pathways

Based on the experimentally validated target genes of miR-204-5p from miRTarBase, we then performed the gene set functional enrichment analysis using the enricher function of the R package clusterProfiler (Yu et al., 2012; Chen et al., 2013) in the Reactome Pathway Database (Jassal et al., 2020). Reactome functions both as an archive of biological processes and as a tool for discovering functional relationships in data such as gene expression profiles (Jassal et al., 2020). Pathways that were specific to cancer or specific to other diseases and very general signaling pathways that contained over 200 genes were excluded from the search due to gene overlap and redundancy.

## Results

The demographic and clinical characteristics of pregnant women who subsequently developed PE and those who remained healthy during pregnancy enrolled in the study are shown in (Table 1). At the time that maternal venous blood samples were collected, all the demographic and clinical characteristics of the subjects included, such as age, body mass index, systolic and diastolic blood pressure, and gestational age at sampling were similar between the study groups. As expected, the mean newborn weight of pregnant women who subsequently developed PE (case group) was significantly lower than that of the healthy pregnant women included in the control group (*P* < 0.05, Table 1).

**Table 1:** Demographic and clinical characteristics of subjects enrolled in the study.

**Parameters**	**Preeclampsia (case group)**	**Healthy pregnant (control group)**	***P***
Number of subjects	5	4	
Age (years)	29.8 ± 2.0	28.8 ± 2.6	0.7541
BMI at sampling (kg/m^2^)	28.6 ± 1.8	28.5 ± 0.7	0.9664
SBP (mmHg) at sampling	115.0 ± 5.7	102.5 ± 3.2	0.1199
DBP (mmHg) at sampling	73.0 ± 6.0	62.5 ± 1.4	0.1755
GA at sampling (weeks)	23.4 ± 0.6	22.0 ± 0.6	0.1429
Newborn weight (g)	1942.0 ± 169.4	3623.0 ± 258.4	0.0008^*^

*BMI, body mass index; SBP, systolic blood pressure; DBP, diastolic blood pressure; GA, gestational age*.

* *P < 0.05 vs. control group*.

*Data as mean ± S.E*.

The expression profile for all the 84 circulating miRNAs assessed in plasma samples from pregnant women who subsequently developed PE (case) and those who remained healthy during pregnancy (control), the fold change values between the groups (case/control), and the *P-*values are shown in Supplementary Table 2. Overall, 23 miRNAs were considered to be upregulated by using the fold change > 2.0 in plasma samples from pregnant women who subsequently developed PE as compared to those who remained healthy during pregnancy. However, only the miR-204-5p had a null hypothesis of significance testing lower than 0.05 (*P* = 0.0082; Table 2). Notably, the miR-24-3p, miR-22-3p, miR-143-3p, and miR-376c-3p showed a fold change > 4.0, which means that their expression was more than four times higher in pregnant women who subsequently developed PE than that in women who remained healthy during pregnancy.

**Table 2:** Fold-change values for the circulating miRNAs expression in plasma from pregnant who developed severe preeclampsia (case group) compared with those who remained healthy during pregnancy (control group).

**miRNAs**	**Fold change (case/control)**	***P***
hsa-miR-24-3p	9.4011	0.4951
hsa-miR-22-3p	5.2683	0.2170
hsa-miR-143-3p	5.0049	0.3910
hsa-miR-376c-3p	4.6649	0.2309
hsa-miR-19b-3p	3.2986	0.1323
hsa-miR-17-5p	3.234	0.1492
hsa-miR-106b-5p	3.1587	0.1021
hsa-miR-15a-5p	3.1576	0.1726
hsa-miR-19a-3p	2.8865	0.1779
hsa-miR-29a-3p	2.7242	0.2728
hsa-miR-128-3p	2.7185	0.4609
hsa-miR-200b-3p	2.6691	0.1673
hsa-miR-146a-5p	2.6296	0.2794
hsa-miR-34a-5p	2.4357	0.2510
hsa-miR-204-5p	2.3823	0.0082^*^
hsa-miR-27a-3p	2.3683	0.4159
hsa-miR-26b-5p	2.3179	0.8274
hsa-miR-223-3p	2.1448	0.8807
hsa-miR-221-3p	2.0681	0.2896
hsa-miR-18a-5p	2.0467	0.2190
hsa-miR-15b-5p	2.0446	0.7782
hsa-miR-103a-3p	2.0249	0.3685
hsa-miR-30e-5p	2.0172	0.6543

*A fold change > 2.0 was used as the cutoff value to consider the upregulated expression of miRNAs as compared with the control group*.

* *P < 0.05 for the miR-204-5p expression in the case group as compared with the control group*.

**Figure 2:**
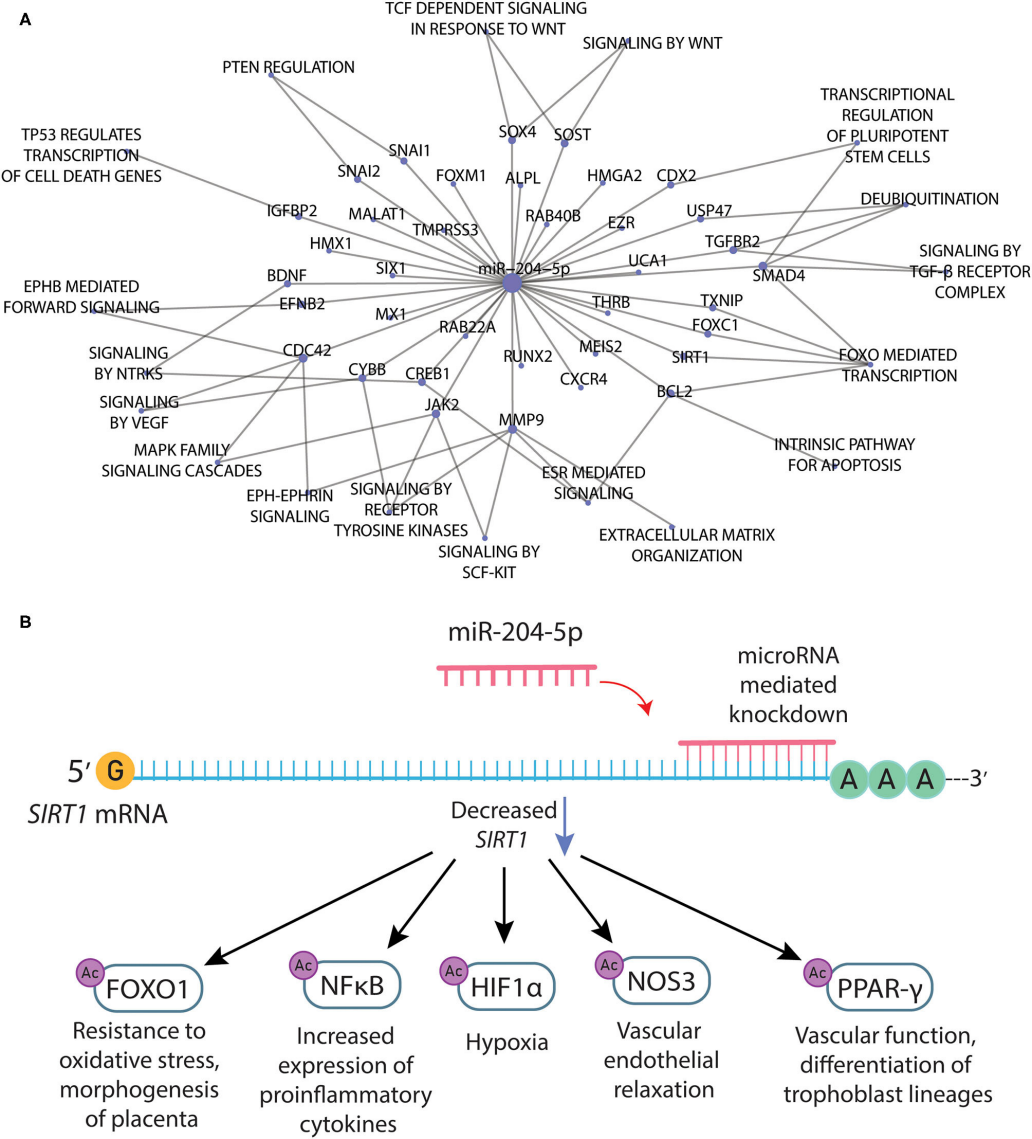
**(A)** Network connecting the target genes and related pathways for the mir-204-5p according to the selection of experimentally validated miRNA-target interactions from miRTarBase. **(B)** Downstream targets of SIRT1 that are related to vascular endothelial function or pathways implicated in the pathophysiology of preeclampsia.

Noteworthy, we searched the miRTarBase for experimentally validated miRNA-target gene interactions for the miR-204-5p, which were used in the functional enrichment to thoroughly explore the network connecting the 37 target genes and their biological effects (Figure 2A). Notably, we found several important genes related to the pathophysiology of PE, such as matrix metallopeptidase 9 (*MMP-9*), metastasis-associated lung adenocarcinoma transcript 1 (*MALAT1*), transforming growth factor beta receptor 2 (*TGFBR2*), and sirtuin-1 (*SIRT1*). Noteworthy, *SIRT1* was found to be downregulated by independent analyses in PE (Viana-Mattioli et al., 2020). Moreover, SIRT1 was shown to have a role in regulating endothelial function, arterial remodeling, and vascular aging (Man et al., 2019). Therefore, we depicted downstream targets of SIRT1 that are related to vascular endothelial function or pathways implicated in the pathophysiology of PE (Figure 2B).

## Discussion

In this study, the main novel findings were that 23 circulating miRNAs were considered to be upregulated by using the fold change > 2.0 in plasma samples collected in the second trimester from pregnant women who subsequently developed PE as compared with those who remained healthy during pregnancy, even before the onset of clinical symptoms of PE. Noteworthy, only the miR-204-5p was statistically significant (*P* = 0.0082).

The search for potential predictive biomarkers is clinically relevant because it enables the early identification of pregnant women at high risk of developing PE, which may allow to take actions to minimize future complications for the mother and fetus. Since most of the cases of PE occur after 25 weeks of gestation, we focused on the period of 20–25 weeks of gestation in order to maximize the chance to find predictive biomarkers to monitor the pregnancy. In this context, several candidates for circulating biomarkers have been studied, such as pregnancy-associated plasma protein-A (PAPP-A), C-reactive protein (CRP), interleukin-1β (IL-1β), antiangiogenic molecules, homocysteine, oxidative stress markers, and vasoactive peptides (Levine et al., 2004; D'antonio et al., 2013; Maged et al., 2017; Rocha-Penha et al., 2017; Lind Malte et al., 2018; Sandrim et al., 2018a,b; Machado et al., 2019). However, most of these biomarkers showed poor to moderate predictive value for PE. Noteworthy, the sFLT-1/PIGF ratio has been shown to be the most promising biomarker, which is the combination of increased levels of the antiangiogenic factor soluble fms-like tyrosine kinase-1 (sFLT-1) along with the decreased levels of placental growth factor (PIGF) (Cerdeira et al., 2019).

The expression profile of circulating miRNAs was reported to be different in pregnant women who subsequently developed PE. However, only some of the miRNAs reported had predictive value for PE (Luque et al., 2014; Hromadnikova et al., 2017, 2019). Notably, miR-143 and miR-221 were found to be upregulated in samples collected in the first trimester (Luque et al., 2014). In this study, we found that 23 miRNAs were considered to have upregulated expression by using the fold change > 2.0 (Zhao et al., 2018) in plasma samples collected in the second trimester from pregnant women who developed PE. Among these, the miR-143-3p, miR-22-3p, and miR-24-3p had a fold change > 4.0 in pregnant women who developed PE, which suggests their role in the early stages of the pathophysiology of PE. Notably, miRNA-143 is essential for the differentiation of vascular smooth muscle cells (Zhao et al., 2015), and its levels were significantly increased in peripheral blood leucocytes from hypertensive patients (Chen et al., 2018). Moreover, miR-22 was found to be upregulated in preeclamptic placentas (Shao et al., 2017), and miR-24 was found to be increased in plasma from patients with severe preeclampsia (Wu et al., 2012). However, only the miR-204-5p was statistically significant (*P* = 0.0082), while the other 22 miRNAs were upregulated but not statistically significant (Table 2).

The functional enrichment of target genes for the upregulated miR-204-5p in the Reactome Pathway Database revealed pathways associated with the pathophysiology of PE (Figure 2A). Noteworthy, miR-204-5p directly binds to transforming growth factor beta receptor 2 (TGFBR2), a part of the TGF-beta-binding complex. Notably, disruption of TGF-beta signaling is a common characteristic in PE, as reviewed elsewhere (Powe et al., 2011) with excess secretion of soluble endoglin and soluble Flt1 (sFlt-1) directly dysregulating this signaling (Wang et al., 2009). miRNAs could be another player acting to dysregulate TGF-beta-related pathways, which could help to explain the endothelial cell dysfunction and decreased nitric oxide production previously found in severe PE (Powe et al., 2011). Moreover, miR-204 was shown to suppress trophoblast invasion by targeting the MMP-9 (Yu et al., 2015), and it was significantly overexpressed in the preeclamptic placenta (Choi et al., 2013). MALAT1, a long noncoding RNA commonly found to be hyperexpressed in cancer (Tian and Xu, 2015), is also essential for trophoblast invasion and proliferation in the early stages of pregnancy (Li et al., 2017, 2020), and it was found to be downregulated in patients with PE. Noteworthy, the miR-204-5p interacts with MALAT1 (Li et al., 2016), which could explain its downregulation in PE patients. However, this hypothesis remains to be tested. Among the target genes for miR-204-5p, there are also several ephrin ligands and signaling proponents, such as ephrin 2 (*EFNB2*) and cell division cycle 42 (*CDC42*). Notably, ephrin-related pathways have a major role in embryogenesis, and other upregulated miRNAs found in preeclamptic placentas were shown to target related genes (Wang et al., 2012). Taken together, these findings indicate that target genes for the miR-204 could also be involved in the early pathogenesis of PE.

The miRNA 204-5p was found to be upregulated in the serum of patients with PE, and its knockdown induced a better survival rate of hypoxic cells (Mei et al., 2017). However, this previous study did not explore the target genes for the miRNA 204-5p nor the mechanisms of action of these target genes. Interestingly, we found that some of the target genes for the miRNA 204-5p were also found to be downregulated by independent analyses in PE, such as brain-derived neurotrophic factor (*BDNF*) (Perucci et al., 2017) and *SIRT1* (Viana-Mattioli et al., 2020). For example, SIRT1 can deacetylate histones and lead to gene silencing, and it can also regulate the function of several target proteins by deacetylation (Gomes et al., 2016). Endothelial SIRT1 is proven to regulate endothelial function, arterial remodeling, and vascular aging (Man et al., 2019) by interacting with a large network of proteins, such as the forkhead box class O family member proteins (FoxOs), the nuclear factor kappa B (NF-κB), tumor protein 53 (p53), hypoxia-inducible factor-1a (HIF-1α), the superoxide dismutase (SODs), the peroxisome proliferator-activated receptor-γ (PPAR-γ) and its coactivator-1α (PGC-1α) (Chong et al., 2012). Therefore, we depicted selected downstream targets of SIRT1 that have implications for the pathophysiology of PE (Figure 2B) and described their biological roles below.

FOXO1 is implicated in the regulation of oxidative stress and apoptosis (Gomes et al., 2016). Moreover, FOXO1 is implicated in the regulation of a variety of other cellular processes that are critical for the placenta, including cell cycle regulation, cellular differentiation and proliferation, DNA repair, and metabolism (Monsalve and Olmos, 2011). Interestingly, FOXO1 expression in the developing mouse embryo was observed to be essential for placental morphogenesis (Ferdous et al., 2011). Since FOXO1 is critical for placental cellular morphogenesis, abnormal FOXO1 expression may contribute in part to the abnormal trophoblast differentiation in mild PE (Sheridan et al., 2015). The NFκB protein family regulates several pathways within the cell-including inflammation, hypoxia, angiogenesis, and oxidative stress, all of which are implicated in placental development (Armistead et al., 2020). Notably, the role of hypoxia and HIF-1α in the pathogenesis of PE, and the possible molecular links between hypoxia and potential mediators of PE pathogenesis are reviewed elsewhere (Tal, 2012). Finally, PPAR-γ plays a predominant role in normal vascular function (Marx et al., 1999) and in the differentiation of trophoblast lineages (Schaiff et al., 2000); it was suggested to play a pivotal role in the progression of a healthy pregnancy and may critically regulate the risk of PE (Mccarthy et al., 2011).

SIRT1 also regulates the endothelial nitric oxide synthase (eNOS), also known as nitric oxide synthase 3 (NOS3), which is activated to produce the vasodilator nitric oxide (NO), upon deacetylation of lysines 496 and 506 in the eNOS calmodulin-binding domain (Mattagajasingh et al., 2007). Noteworthy, previous studies proposed that SIRT1 and eNOS are synergistically regulated through an eNOS-NO-SIRT1 positive feedback mechanism that is considered crucial for maintaining regular endothelium function (Ota et al., 2010; Man et al., 2019). Therefore, reduced SIRT1 may play an important role in PE and was shown to contribute to vascular endothelial dysfunction with aging via modulation of eNOS (Ota et al., 2007; Donato et al., 2011). Interestingly, it was also suggested that SIRT1 activation might be related to reduced sFlt-1 secretion in preeclamptic placentas (Cudmore et al., 2012) and trophoblasts (Hannan et al., 2017; Hastie et al., 2019), thereby reducing the effects of this antiangiogenic molecule and improving vascular dysfunction in PE.

This study has limitations. The power of the study regarding the small sample size may have limited our chance to find statistical differences in the expression of miRNAs. Moreover, we did not perform the validation of the miRNAs considered to be differently expressed in a larger quantity of samples using a different experimental method. Therefore, our findings should be replicated on a larger or secondary population. Importantly, further validation studies are needed to investigate the circulating miRNAs identified in our screening based on a prospective cohort of pregnant women as potential predictive biomarkers in PE. However, since the literature is scarce, our findings provide evidence suggesting that miRNAs found to be upregulated in the second trimester from pregnant women who subsequently developed PE could be prioritized as potential candidates by further validation studies focused on establishing predictive biomarkers in PE.

## Conclusion

In conclusion, 23 circulating miRNAs had a fold change > 2.0 and were considered to be upregulated in plasma samples collected between 20 and 25 weeks from pregnant women who subsequently developed PE, even before the onset of clinical symptoms of PE. However, only the miR-204-5p was statistically significant (*P* = 0.0082), which has target genes associated with pathways of known pathophysiological relevance during the early development of PE. Therefore, our novel findings provide for circulating miRNAs identified in the second trimester of pregnancy that may guide further studies focused on the validation of potential predictive biomarkers in PE.

## Data Availability Statement

The authors acknowledge that the data presented in this study must be deposited and made publicly available in an acceptable repository, prior to publication. Frontiers cannot accept a article that does not adhere to our open data policies.

## Ethics Statement

The studies involving human participants were reviewed and approved by Institutional Review Board of the University of São Paulo (USP) at Ribeirão Preto (reference 4116/2008), State of São Paulo, Brazil, and all participants provided written informed consent. The patients/participants provided their written informed consent to participate in this study.

## Author Contributions

ML, IC, MC-D, RC, and VS have made substantial contributions to the conception of the work. ML, IC, SV-M, MC-D, and VS have drafted the manuscript and prepared the figures. ML, IC, SV-M, MC-D, RC, and VS have revised and approved the final version of the manuscript for submission. All authors contributed to the article and approved the submitted version.

## Funding

This work was supported by the National Council for Scientific and Technological Development (CNPq/Brazil) (Grant #2014-5/305587 and #312599/2019-6), the São Paulo Research Foundation (FAPESP/Brazil) (Grants #2008/53593-0, and #2015/20461-8), and the Coordination for the Improvement of Higher Education Personnel (CAPES/Brazil) (Finance Code 001).

## Conflict of Interest

The authors declare that the research was conducted in the absence of any commercial or financial relationships that could be construed as a potential conflict of interest.

## Publisher's Note

All claims expressed in this article are solely those of the authors and do not necessarily represent those of their affiliated organizations, or those of the publisher, the editors and the reviewers. Any product that may be evaluated in this article, or claim that may be made by its manufacturer, is not guaranteed or endorsed by the publisher.

## Supplementary Material

Supplementary Table 1 miRTarBase information on the validated target genes for hsa-miR-204-5p, which were verified by functional miRNA–target interactions and experiments (almost all of them by luciferase reporter assays) performed by manually curated articles (referenced by the respective PubMed ID (PMID).

Supplementary Table 2 Expression profile of 84 circulating miRNAs in plasma samples from pregnant who subsequently developed PE (case group) compared with women who remained healthy during pregnancy (control group). Fold-change values > 2.0 for the upregulated miRNAs in pregnant who subsequently developed PE are highlighted in bold. ^*^miR-204-5p *P* < 0.05 as compared with the expression in the control group.
