# Supplementary material for: Circulating MicroRNAs in the Second Trimester From Pregnant Women Who Subsequently Developed Preeclampsia: Potential Candidates as Predictive Biomarkers and Pathway Analysis for Target Genes of miR-204-5p
Source: Front Physiol. 2021 Sep 22;12:678184. doi: 10.3389/fphys.2021.678184 (PMC8493119; doi:10.3389/fphys.2021.678184)
Supplement: Supplementary Table 2 — Expression profile of 84 circulating miRNAs in plasma samples from pregnant who subsequently developed PE (case group) compared with women who remained healthy during pregnancy (control group). Fold-change values > 2.0 for the upregulated miRNAs in pregnant who subsequently developed PE are highlighted in bold. *miR-204-5p P < 0.05 as compared with the expression in the control group. [file Table_2.DOCX]

**Supplementary Table 2.** Expression profile of 84 circulating miRNAs in plasma samples from pregnant who developed PE (case group) compared to women who remained healthy during pregnancy (control group). The fold change values > 2.0 for the upregulated miRNAs in pregnant who developed PE **are highlighted in bold**. ***miR-204-5p** *P* value < 0.05 as compared to the expression in the control group.

| **miRNA** | **Fold Change (case/control)** | ***P* value** | **miRNA** | **Fold Change (case/control)** | ***P* value** |
| --- | --- | --- | --- | --- | --- |
| hsa-let-7a-5p | 1.7331 | 0.8666 | **hsa-miR-27a-3p** | **2.3683** | 0.4159 |
| hsa-miR-1-3p | 1.8991 | 0.4534 | hsa-miR-296-5p | 0.4036 | 0.3051 |
| hsa-miR-100-5p | 1.7701 | 0.7201 | **hsa-miR-29a-3p** | **2.7242** | 0.2728 |
| **hsa-miR-106b-5p** | **3.1587** | 0.1021 | hsa-miR-30d-5p | 1.3787 | 0.9591 |
| hsa-miR-10b-5p | 1.0114 | 0.3579 | **hsa-miR-34a-5p** | **2.4357** | 0.2510 |
| hsa-miR-122-5p | 0.5609 | 0.2414 | hsa-miR-375 | 0.941 | 0.4568 |
| hsa-miR-124-3p | 0.6703 | 0.2419 | hsa-miR-423-5p | 0.9281 | 0.4085 |
| hsa-miR-125b-5p | 1.681 | 0.8582 | hsa-miR-499a-5p | 1.2577 | 0.6727 |
| hsa-miR-126-3p | 1.7403 | 0.8809 | hsa-miR-574-3p | 0.7246 | 0.3726 |
| hsa-miR-133a-3p | 1.823 | 0.2756 | hsa-miR-885-5p | 0.4301 | 0.2745 |
| hsa-miR-133b | 0.5294 | 0.9634 | hsa-miR-9-5p | 1.2895 | 0.7541 |
| hsa-miR-134-5p | 1.2065 | 0.5565 | hsa-miR-92a-3p | 0.9074 | 0.4276 |
| hsa-miR-141-3p | 0.8443 | 0.5597 | hsa-miR-93-5p | 1.4685 | 0.7180 |
| **hsa-****miR-143-3p** | **5.0049** | 0.3910 | hsa-let-7c-5p | 1.6927 | 0.8475 |
| **hsa-miR-146a-5p** | **2.6296** | 0.2794 | hsa-miR-107 | 1.525 | 0.3856 |
| hsa-miR-150-5p | 1.3612 | 0.5936 | hsa-miR-10a-5p | 1.033 | 0.4541 |
| hsa-miR-155-5p | 0.6983 | 0.4086 | **hsa-miR-128-3p** | **2.7185** | 0.4609 |
| **hsa-miR-17-5p** | **3.234** | 0.1492 | hsa-miR-130b-3p | 1.2766 | 0.6275 |
| hsa-miR-17-3p | 1.1199 | 0.4948 | hsa-miR-145-5p | 1.907 | 0.3827 |
| **hsa-miR-18a-5p** | **2.0467** | 0.2190 | hsa-miR-148a-3p | 1.767 | 0.7220 |
| hsa-miR-192-5p | 1.141 | 0.7176 | **hsa-miR-15a-5p** | **3.1576** | 0.1726 |
| hsa-miR-195-5p | 1.6516 | 0.9815 | hsa-miR-184 | 0.1940 | 0.2370 |
| hsa-miR-196a-5p | 0.3506 | 0.1827 | hsa-miR-193a-5p | 0.7317 | 0.3269 |
| **hsa-miR-19a-3p** | **2.8865** | 0.1779 | **hsa-****miR-204-5p** | **2.3823** | **0.0082*** |
| **hsa-miR-19b-3p** | **3.2986** | 0.1323 | hsa-miR-206 | 0.8723 | 0.3565 |
| hsa-miR-200a-3p | 0.5935 | 0.3188 | hsa-miR-211-5p | 0.7494 | 0.8490 |
| **hsa-miR-200b-3p** | **2.6691** | 0.1673 | **hsa-miR-26b-5p** | **2.3179** | 0.8274 |
| hsa-miR-200c-3p | 1.7548 | 0.3670 | **hsa-miR-30e-5p** | **2.0172** | 0.6543 |
| hsa-miR-203a-3p | 0.4415 | 0.3233 | hsa-miR-372-3p | 0.8127 | 0.6396 |
| hsa-miR-205-5p | 1.1518 | 0.5614 | hsa-miR-373-3p | 0.6093 | 0.6242 |
| hsa-miR-208a-3p | 1.5744 | 0.4163 | hsa-miR-374a-5p | 1.8517 | 0.7398 |
| hsa-miR-20a-5p | 1.9064 | 0.4278 | **hsa-****miR-376c-3p** | **4.6649** | 0.2309 |
| hsa-miR-21-5p | 1.7985 | 0.6291 | hsa-miR-7-5p | 1.0507 | 0.4328 |
| hsa-miR-210-3p | 0.6673 | 0.9743 | hsa-miR-96-5p | 0.4756 | 0.2820 |
| hsa-miR-214-3p | 1.4715 | 0.1493 | **hsa-miR-103a-3p** | **2.0249** | 0.3685 |
| hsa-miR-215-5p | 0.6365 | 0.3118 | **hsa-miR-15b-5p** | **2.0446** | 0.7782 |
| **hsa-miR-221-3p** | **2.0681** | 0.2896 | hsa-miR-16-5p | 1.5826 | 0.9950 |
| hsa-miR-222-3p | 1.5287 | 0.2168 | hsa-miR-191-5p | 1.8762 | 0.5227 |
| **hsa-miR-223-3p** | **2.1448** | 0.8807 | **hsa-****miR-22-3p** | **5.2683** | 0.2170 |
| hsa-miR-224-5p | 0.7707 | 0.3451 | **hsa-****miR-24-3p** | **9.4011** | 0.4951 |
| hsa-miR-23a-3p | 1.9057 | 0.5821 | hsa-miR-26a-5p | 1.925 | 0.9151 |
| hsa-miR-25-3p | 1.3207 | 0.5949 | hsa-miR-31-5p | 1.1288 | 0.8585 |
